# Trichilemmal Keratosis of the Lower Leg: A Case Report

**DOI:** 10.7759/cureus.113200

**Published:** 2026-07-22

**Authors:** Mariam N Albrahim, Tareq Mohammad, Qutaibah Alsahly, Ali Naqi, Abdullah Alkhars

**Affiliations:** 1 Department of Histopathology, Jaber AlAhmad Hospital, Kuwait City, KWT; 2 Dermatology, Mubarak Al Kabeer Hospital, Jabriya, KWT

**Keywords:** cutaneous horn, follicular keratinzation, trichilemmal horn, trichilemmal keratinization, trichilemmal keratosis

## Abstract

Trichilemmal keratosis is an underreported follicular keratinizing lesion that arises from the outer root sheath of the hair follicle. It is frequently confused with other common keratotic lesions. We report a 49-year-old female patient who presented with an asymptomatic nodular lesion with a prominent keratinous center on the lower dorsal aspect of the left leg. Histopathological evaluation confirmed the diagnosis of trichilemmal keratosis. This case highlights the characteristic clinical presentation of trichilemmal keratosis and its mimickers, reinforcing the importance of careful assessment of patient history, lesion morphology, and growth pattern in guiding diagnostic evaluation.

## Introduction

Trichilemmal keratosis, or trichilemmal horn, is a potentially underrecognized morphologic growth pattern that arises from the outer root sheath of the hair follicle and is characterized by abrupt trichilemmal maturation of keratinocytes in the absence of a granular layer [1]. It represents a proliferation of follicular epithelium with trichilemmal differentiation [2]. Clinically, it forms a horn-like, compact vertical column of keratin extending from a follicular structure. This lesion shows a female predominance, accounting for approximately two-thirds of reported cases [2,3]. While trichilemmal keratosis has been described in patients aged 16 to 83 years, it is most frequently seen in individuals over 50 years of age [3]. Trichilemmal keratosis may resemble a cutaneous horn arising from premalignant or malignant epidermal lesions such as seborrheic keratosis, hypertrophic actinic keratosis (HAK), keratoacanthoma, and squamous cell carcinoma. However, attention to the clinical context, including patient age, lesion location, and growth pattern, may provide useful diagnostic clues. Accurate recognition is important because trichilemmal keratosis may be mistaken for premalignant or malignant keratinizing lesions, potentially leading to unnecessary treatment. We report this case to highlight the diagnostic challenge and emphasize the inclusion of trichilemmal keratosis in the differential diagnosis of hyperkeratotic cutaneous lesions.

## Case presentation

A 49-year-old female patient presented to the outpatient department of dermatology with an asymptomatic nodular lesion that measured approximately 1.5 cm and had a keratinous center on the lower extensor aspect of the lower leg (Figure 1).

**Figure 1:**
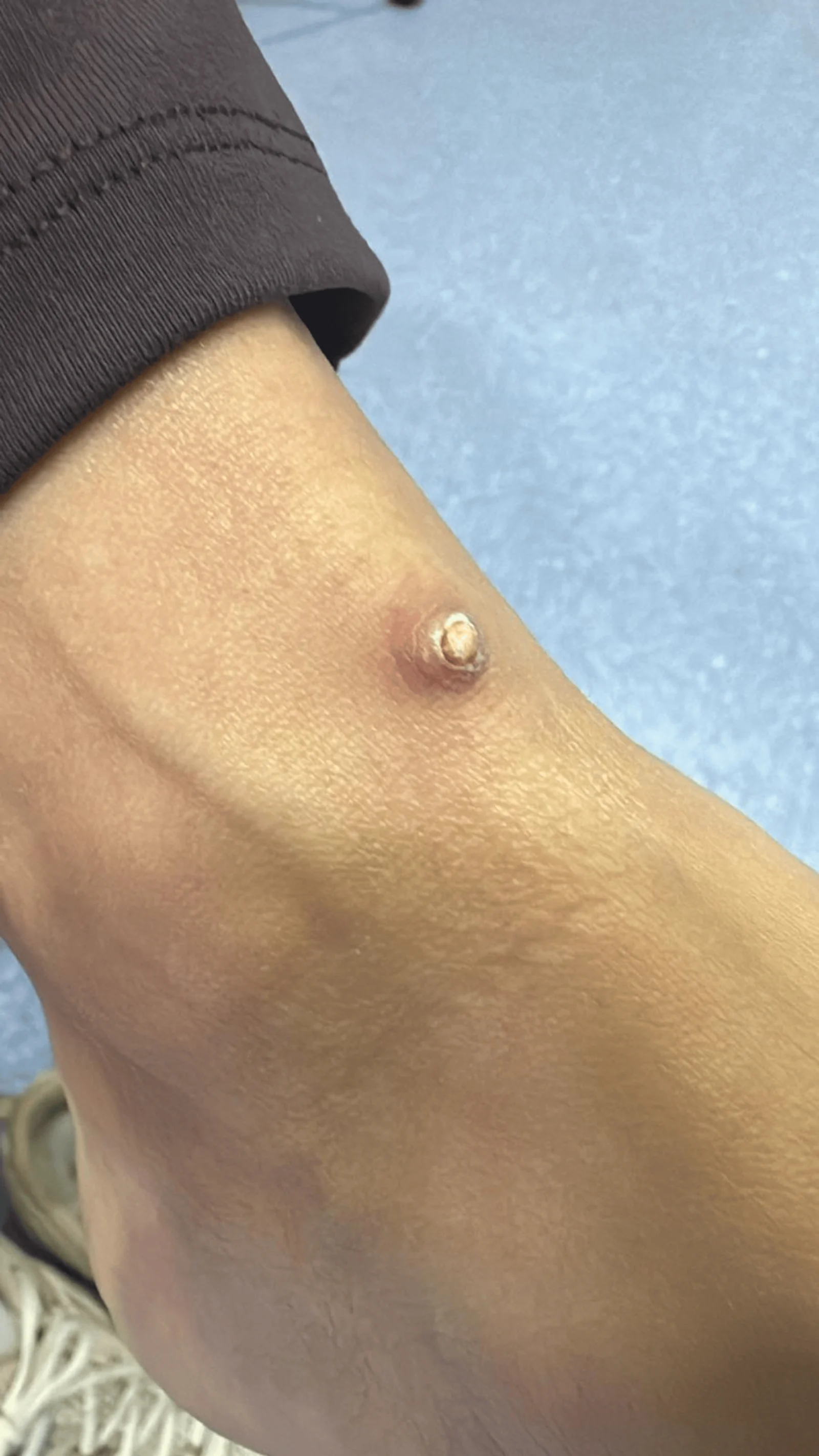
Clinical appearance of trichilemmal keratosis A well-circumscribed hyperkeratotic nodule with a central keratin plug on the distal extensor aspect of the lower leg.

The lesion had gradually enlarged before presentation and was not associated with any preceding trauma or local procedures. On physical examination, a firm nodular lesion with a central keratinous plug was noted. The clinical differential diagnosis included keratoacanthoma and calcinosis cutis. An excision was performed. The postoperative course was uneventful, with satisfactory healing and no evidence of recurrence during the available clinical follow-up. However, the exact duration of follow-up was not documented in the medical records. Histological examination revealed a crateriform stratified squamous epithelium with a keratotic center extending through the reticular dermis (Figure 2).

**Figure 2:**
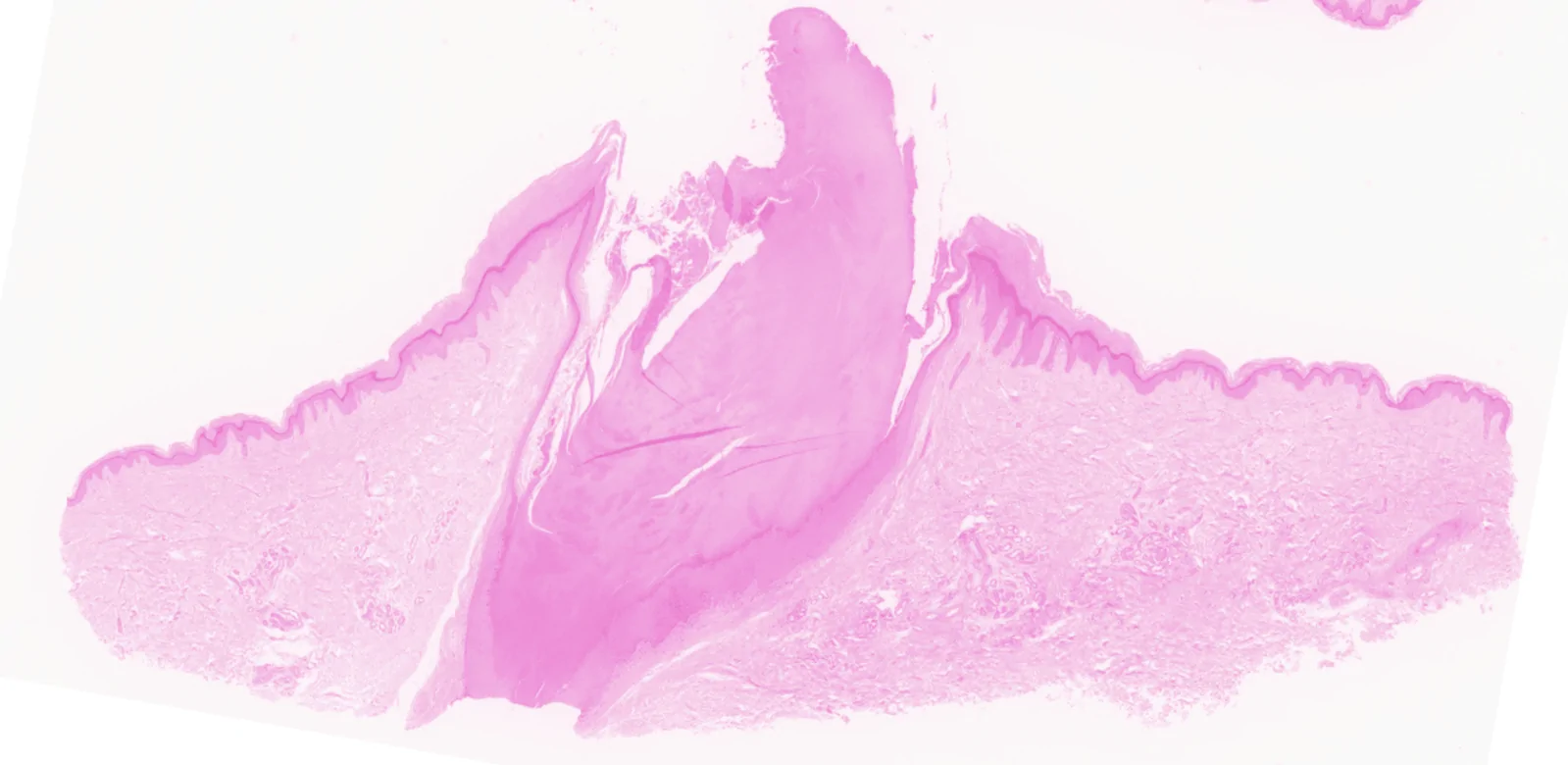
Histological appearance of trichilemmal keratosis showing crateriform stratified squamous epithelium with a keratotic center, (H&E, x2.5) H&E: hematoxylin and eosin

The squamous epithelium showed subtle lobulation with abrupt trichilemmal-type keratinization and a limited residual granular layer, in addition to peripheral palisading of the outer layer (Figures 3, 4).

**Figure 3:**
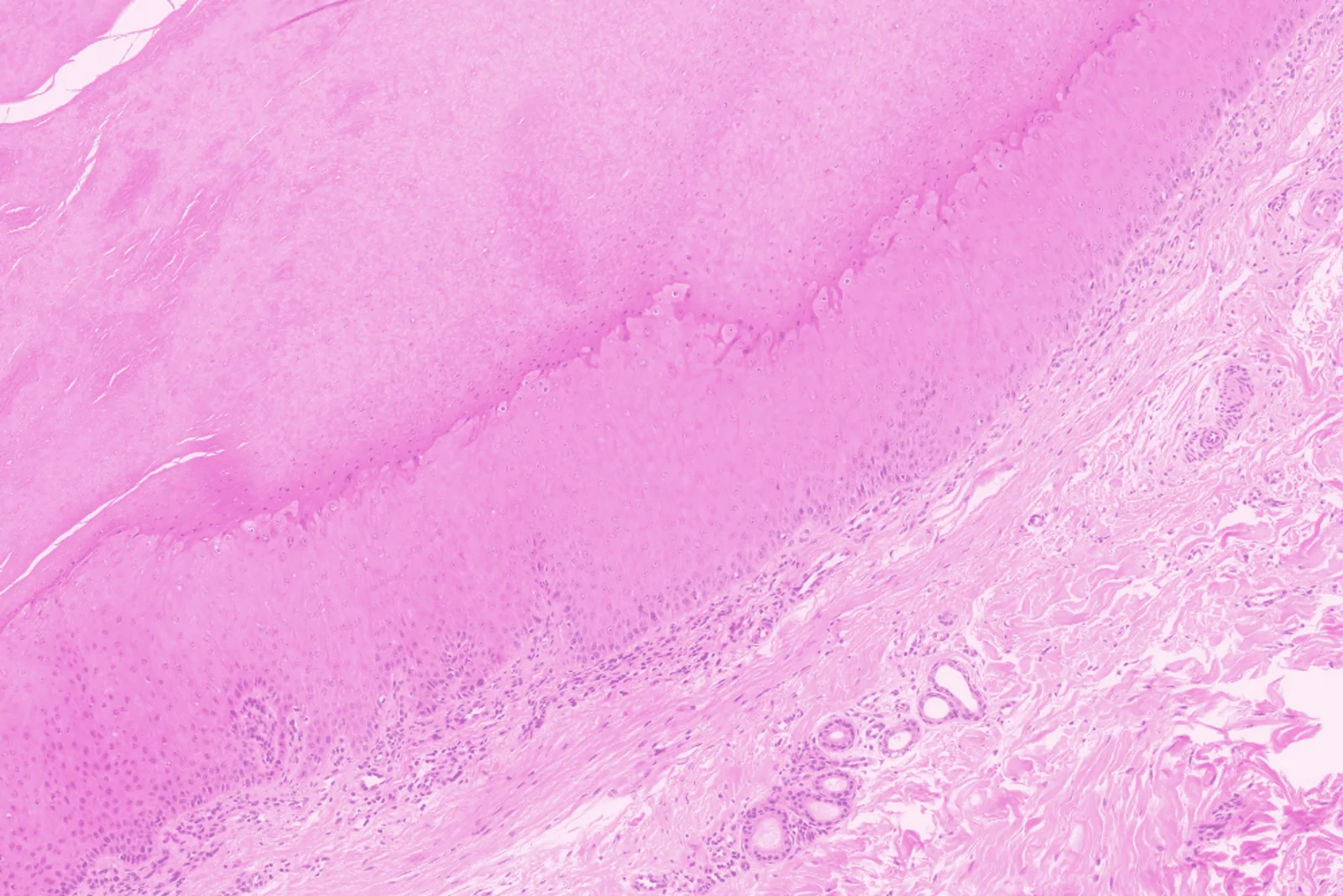
Medium view showing squamous epithelium with subtle lobulation and abrupt trichilemmal-type keratinization, (H&E, x100) H&E: hematoxylin and eosin

**Figure 4:**
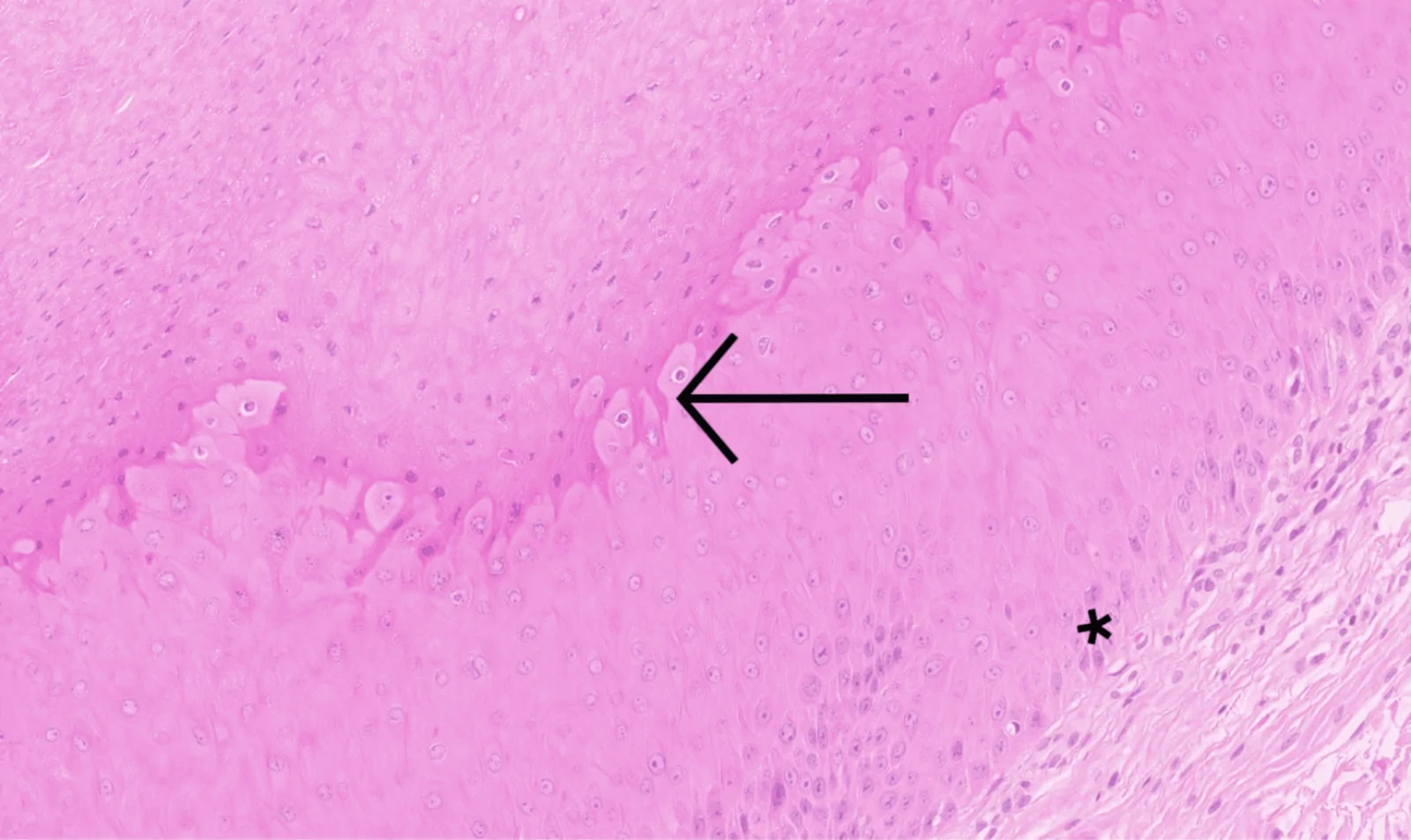
High power showing trichilemmal keratinization (H&E, x250) Arrow: abrupt keratinization and absence of granular layer; Asterisk: peripheral palisading H&E: hematoxylin and eosin

The crater contained dense eosinophilic keratinous material with ‘horn-like’ exophytic extension beyond the crater’s opening.

## Discussion

Trichilemmal keratosis is a benign follicular keratinizing lesion of the skin. On clinical examination, it typically occurs in hair-bearing areas, particularly the scalp and face. Ectopic cases involving sites such as the palm and thumb have also been described [4,5]. In the present case, the patient presented with a lesion on the distal extensor aspect of the lower leg, further demonstrating that trichilemmal keratosis may occur outside its more frequently described locations. Regarding patient demographics, trichilemmal keratosis can occur at any age but shows a predilection for middle-aged individuals [2]. Our patient's age, 49 years, was consistent with this reported pattern. Clinically, trichilemmal keratosis is usually solitary and well-circumscribed, with a firm base and a prominent central keratinous component that appears compact, dense, and vertically oriented [3,4]. The surface may be smooth, laminated, or mildly verrucous, and the lesion often gives the impression of arising from a follicular unit. These lesions are typically asymptomatic and may remain stable for prolonged periods or gradually enlarge over time [3]. The lesion in our patient followed this pattern, remaining asymptomatic while slowly increasing in size. The clinical evaluation should include assessment for a pre-existing pilar/trichilemmal cyst, prior manipulation (e.g., picking, shaving, friction), and previous local procedures (e.g., incision and drainage, excision, cautery), as these factors have been reported in association with trichilemmal keratosis [6,7]. Histologically, trichilemmal keratosis is characterized by marked orthokeratotic eosinophilic hyperkeratosis, verrucous epidermal hyperplasia, an absent granular layer, and abrupt trichilemmal keratinization [1]. The keratinocytes transition directly into compact lamellar keratin in a pattern resembling that of the follicular isthmus and trichilemmal cysts [1,2]. The superficial dermis may show epithelial lobules composed of large, pale-staining keratinocytes with peripheral basal palisading and centripetal keratinization [1]. Sporadic secondary germinative buds may arise from the peripheral lobular epithelium, while deeper lobules may resemble those seen in proliferating trichilemmal tumors [1,2]. In our case, the absence of a granular layer together with abrupt trichilemmal keratinization supported the diagnosis.

The principal differential diagnoses of trichilemmal keratosis include seborrheic keratosis, hypertrophic actinic keratosis, keratoacanthoma, squamous cell carcinoma in situ, and invasive squamous cell carcinoma. Seborrheic keratosis is distinguished clinically by its “stuck-on” appearance and histologically by basaloid proliferation with horn cysts [8,9]. Hypertrophic actinic keratosis (HAK) typically occurs on chronically sun-exposed skin and shows keratinocyte atypia, parakeratosis, and solar elastosis, with retention of the granular layer [2,10]. Keratoacanthoma (KA) is characterized by rapid growth and symmetric crateriform architecture with a central keratin plug [11,12]. In contrast, squamous cell carcinoma in situ (SCCIS) demonstrates full-thickness epidermal atypia without dermal invasion, whereas invasive squamous cell carcinoma (SCC) shows cytologic atypia, mitotic activity, and infiltrative growth [12,13]. Other lesions that may present as a cutaneous horn include lichenoid keratosis, basal cell carcinoma, adnexal neoplasms such as eccrine poroma, and metastatic disease [14-16]. In the present case, the lower-leg location and central keratinous component prompted consideration of these keratinizing lesions; however, the histopathologic features favored trichilemmal keratosis and excluded malignant mimickers. The principal clinical and histopathologic differential diagnoses are summarized in Table 1.

**Table 1:** Clinical and histopathological features of the principal differential diagnoses of trichilemmal keratosis

Lesion	Typical clinical features	Key histopathological features
Trichilemmal keratosis	Solitary hyperkeratotic papule or nodule, usually occurring on hair-bearing skin but may occur at ectopic sites	Lobular squamous proliferation with abrupt trichilemmal keratinization, absent granular layer, and peripheral palisading; no significant cytologic atypia
Seborrheic keratosis	Well-circumscribed verrucous or "stuck-on" papule or plaque	Acanthosis, papillomatosis, hyperkeratosis, and horn cysts composed predominantly of basaloid keratinocytes
Hypertrophic actinic keratosis (HAK)	Rough, scaly papule or plaque occurring mainly on chronically sun-exposed skin	Basal keratinocyte atypia, disordered epidermal maturation, parakeratosis, and solar elastosis
Keratoacanthoma (KA)	Rapidly growing, dome-shaped nodule with a central keratin-filled crater	Symmetrical crateriform architecture with a central keratin plug and well-differentiated squamous cells with characteristic epithelial "lipping"
SCC in situ (SCCIS)	Slowly enlarging scaly plaque	Full-thickness epidermal atypia without dermal invasion
Squamous cell carcinoma (SCC)	Enlarging, indurated, hyperkeratotic or ulcerated papule, plaque, or nodule	Invasive atypical squamous cells, pleomorphism, mitotic activity, and keratin pearl formation	

The exact pathogenesis of trichilemmal keratosis remains incompletely understood, and current knowledge is largely based on clinicopathologic observations rather than established molecular mechanisms. One proposed mechanism is that excessive keratin production relative to surface shedding leads to the vertical accumulation of compact lamellar keratin along the follicular axis and subsequent formation of a horn-like projection [2]. Several factors have been suggested as possible triggers, although no definitive association has been established. Reported associations include prior trauma or surgical manipulation of a trichilemmal (pilar) cyst, suggesting that disruption of follicular architecture may promote altered trichilemmal differentiation [3]. In addition, trichilemmal keratosis has been described in older patients and on sun-exposed skin, raising the possibility of a relationship with cumulative ultraviolet (UV) exposure and age-related follicular changes. Pre-existing follicular lesions, including trichilemmal cysts and focal outer root sheath hyperplasia, may also provide a substrate for trichilemmal keratinization [3]. Human papillomavirus (HPV) has also been considered in isolated reports following the identification of intranuclear inclusion bodies morphologically similar to HPV on electron microscopic study; however, its role remains speculative [17].

Available reports do not establish standardized management or surveillance recommendations for trichilemmal keratosis, and evidence regarding recurrence and malignant potential remains limited [1-7]. In the present case, complete excision permitted histopathologic diagnosis and removal of the lesion. The patient subsequently demonstrated satisfactory wound healing with no reported recurrence; however, the duration of follow-up was unavailable.

## Conclusions

This case of trichilemmal keratosis contributes to the limited dermatologic literature describing this uncommon follicular keratinizing lesion and highlights its clinical relevance. Trichilemmal keratosis may present with features that overlap with a wide range of benign, premalignant, and malignant lesions. Therefore, although careful clinical assessment is essential, histopathologic examination remains the diagnostic gold standard for establishing the correct diagnosis and preventing misclassification. Awareness of this entity may help clinicians include trichilemmal keratosis in the differential diagnosis of hyperkeratotic cutaneous lesions and avoid unnecessary concern for malignancy.
